# A study of ^131^iodine-labeling of histamine-indomethacin: its in vivo therapeutic effect and anti-tumor mechanisms in Lewis-bearing lung cancer

**DOI:** 10.1186/1748-717X-8-74

**Published:** 2013-03-26

**Authors:** GuoXiu Lu, Guoxu Zhang, CaiXia Zhang, Chunmei Chen, Ruihao Liu

**Affiliations:** 1 Department of Nuclear Medicine, The General Hospital of Shenyang Military Area Command, No 83, Wenhua Road, Shenhe District, Shenyang, 110840, China; 2 Department of Nuclear Medicine, The Shengjing Hospital of China Medical University, Shenyang, 110004, China; 3 Department of Radiology, Longnan Hospital of Daqing, Heilongjiang, China; 4 Department of Cardiovasology, An gang General Hospital-the Eighth Affiliated Hospital of China Medical University, Anshan, Liaoning, China

## Abstract

**Background:**

In our research,we study the effect of ^131^iodine-labeled histamine-indomethacin (^131^I-His-IN). We focus on its in vivo therapeutic effect and anti-tumor mechanisms in Lewis-bearing lung cancer.

**Methods:**

^131^I-His-IN was administered by garage to the mice. At different timepoints, we made autoradiography (ARG) slices to observe the distribution of ^131^I-His-IN in the cellular, and the sliced samples underwent hematoxylin and eosin (HE) staining for observation of tumor necrosis. Before treatment, the groups of mice underwent ^18^F-fluorodeoxyglucose (^18^F-FDG) positron emission tomography-computed tomography (PET-CT) scans ,and they were then given physiologic saline, iodine ^131^ (^131^I), indomethacin (IN), Histamine-indomethacin (His-IN), and ^131^I-His-IN, respectively, three times daily for seven days. Seven days later, all the mice underwent ^18^F-FDG PET-CT scans again. We calculated the maximum standard uptake value (SUVmax) of the region of interest (ROI) and tumor inhibition rate at the same time.

**Results:**

In ARG groups, black silver particle was concentrated in the nucleus and cytoplasm. ^131^I-His-IN mainly concentrated in tumor tissues. At 8 hours after ^131^I-His-IN, the radioactivity uptake in tumor tissue was higher than in other organs (F=3.46,P<0.05). For the ^18^F-FDG PET-CT imaging, the tumor tissus’es SUVmax of the ROI was lower compared to other groups after the treatment with ^131^I-His-IN. The tumor inhibitory rate (54.8%) in ^131^I-His-IN group was higher than in other groups, too. In the ^131^I-His-IN group the vascular endothelial growth factor (VEGF) decreased gradually compared to other groups. The tumor tissue necrotized obviously in ^131^I-His-IN group.

**Conclusions:**

Through these animal experiments, we found ^131^I-His-IN could inhibit the Lewis lung cancer cells. ^131^I-His-IN focused at the cell nucleus and cytoplasm. It could reduce VEGF and increase tumor inhibitory rate. At the same time, ^18^F-FDG PET-CT scan could be used for a curative effect and monitoring of disease prognosis.

## Background

Lung cancer is one of the most common malignant tumors and is associated with a high mortality. The 5-year survival rate after diagnosis is less than 13%, so early and accurate diagnosis and appropriate treatment are key factors in the prognosis of the disease. However, conventional chemotherapy and radiotherapy do not always produce ideal results. A number of clinical and pharmacodynamic studies have reported that indomethacin, a nonsteroidal anti-inflammatory drug (NSAID) with antirheumatic, antipyretic, and analgesic effect. It also has certain antitumor effect. Studies have shown indomethacin (IN) could strongly affinity and selectively accumulate in the tumor tissue. In recent years, some studies have successfully used a nuclide as a tumor marker for labeling of anti-tumor agents. The use of iodine^131^ (^131^I) radionuclide for labeling of indomethacin has been rarely reported and its mechanism is still under study. This study explores the mechanism of action and treatment effects of ^131^I-histamine-indomethacin (^131^I-his-IN) on Kunming mice inoculated with Lewis lung cancer cells.

## Methods

### Animals

The present study was carried out in the Experimental Research Center, Sheng Jing Hospital of China Medical University Hospital, Shenyang, China, between December 2010 and November 2011. The study was approved by the Experimental Animal Committee of Sheng Jing Hospital of China Medical University Hospital. Mouse (n=6) Lewis lung cancer cells were obtained from the China Academy of Medical Sciences, Institute of Materis Medica, Beijing, China. Seventy-five Kunming mice (age 6–8 weeks; weight 20 ± 2 g) were purchased from the Laboratory animal center, Shengjing Hospital of China Medical University.

### Main regents and instruments

Indomethacin, with a purity of 98.5%, was obtained from YunPeng Linfen Shanxi pharmaceutical company. Histamine-indomethacin (His-IN), with a purity of >95%, was obtained from Shenyang Pharmaceutical University. ^131^I-his-IN was prepared using the method by Cai Jiubo et al [1], with a purity of 95% and a specific activity >185 GBq/g. Nuclear emulsion nuclear-4 was purchased from the Institute of Modern Physics, Shanxi Normal University.

### In vivo mouse models with Lewis-bearing lung cancer and groups

Lewis-bearing lung cancer mice were sacrificed when the tumor size reached 1 to 1.5 cm in diameter. Growth cell tumor tissue (10 g) was selected for grounding, and cut into small pieces. The cell suspension was then diluted with 30 mL of physiological saline. Seventy-five Kunming mice were inoculated by hypodermic needle at the axillary skin of right forelimb with 0.2 mL of the cell suspension containing approximately 2 × 10^6^ cells. When the tumor reached a maximum of 1.5 to 2.0 cm in diameter, 75 of the Lewis-bearing lung cancer cell Kunming mice were randomly divided into 15 groups with 5 mice in each group. Each mouse was injected intravenously at the tail with 2 mL 0.1% potassium iodide within 3 days before inoculation in order to block the thyroid gland. This procedure was done for 7 days, three times per day.

### Autoradiography (ARG) groups

Fifty of the Lewis-bearing lung cancer Kunming mice were then divided into 10 groups randomly, with 5 mice in each group. One group served as the control and was injected with 0.2 mL physiological saline (group A), The others 9 groups (groups B-J) were injected intravenously with I^131^-His-IN at a dosage of 370 KBq (0.2 mL) at the tail. After the injection, at different timepoint (B, 0.5 h; C, 1 h; D, 2 h; E, 4 h; F, 8 h; G, 12 h; H, 24 h; I, 48 h; and J, 72 h), samples of ocular blood (0.2-mL) were collected under anesthesia prior to sacrificing the animals. Samples of the liver, kidney, blood, and muscles were collected, washed with physiological saline, and dried by filter paper. Those samples were then weighed. The radioactivity was measured using an R-well type counter instrument. The percentage of radioactive counts per gram of tissue (%ID) and the ratio of tumor tissue to nontumor tissue were calculate.

The tumor tissue was made into ARG slices and HE stained slices. For preparation of the ARG slices, tissue was stored at 4°C for 24 hours. The slices were then developed and fixed, and HE dyed, then we observed the distribution of silver particles with optical microscope.

### ^18^F-FDG Positron emission tomography-computed tomography (PET-CT) imaging

Twenty-five of the Lewis-bearing lung cancer cell Kunming mice were divided into 5 groups randomly respectively (5 mice in each group). They were: K (I^131^ group); L (IN group); M, (His-IN group); N (I^131^-His-IN group), and O (physiological saline as the control group). All the mice were injected with 3.7 MBq/200 μL of ^18^F-FDG into the tail intravenously. They were scanned with ^18^F-FDG Positron emission tomography-computed tomography (PET-CT) (GE Discovery DST 16 PET-CT,USA). Then the test groups( from K to N) were treated with different drug. For group K, ^131^I was injected locally into the tumor site; Others the drug was instilled into the stomach. The dosages for each group were: 0.2 mL of 370 KBq for the ^131^I group, 0.4 mg/kg for the IN group, 0.4 mg/kg for the His-IN group, 0.2 mL of 370 KBq for the ^131^I-His-IN group, and 0.2 mL of physiological saline for the control group. This was carried out for 7 days three times per day. 2 days after treatment, the mice underwent ^18^F-FDG PET-CT imaging again.

To standardize imaging conditions and assure blood glucose levels mice were fasted for at least 6 hours prior to ^18^F-FDG-PET-CT imaging. The CT acquisition parameters were 130 kVp, 100 mAs, 1 second rotation, and 4 mm slice collimation at 8 mm/s bed speed. Approximately 60 minutes before imaging, mice were injected intravenously with 3.7 MBq/200 μL of ^18^F-FDG. The duration of PET emission scan per bed position varied between 1 and 5 minutes as previously described. Care was taken to assure a similar time interval from the injection of FDG to the start of imaging for baseline and follow-up studies. The CT images were reconstructed using conventional filtered back-projection, at 3.4 mm axial intervals to match the slice separation of the PET data. PET images were reconstructed using iterative algorithms Ordered Subset Expectation Maximization (OSEM two iterations, eight subsets). To correct for photon attenuation, a previously published CT-based algorithm was applied.

Image Analysis: The Mirada workstation (REVEALMVS; CTI Mirada Solutions, GE, USA) was used to view PET and CT images. We also measured the FDG uptake of tumor tissue. All FDG images were analyzed by two observers who were blinded to histopathological treatment response. The single maximum pixel value within the slice with the highest radioactivity concentration was detected on baseline and follow-up scans as previously described. The maximum standardized uptake value (SUVmax) was calculated by dividing the activity in that pixel by the administered dose normalized to mice body weight (g/mL). One radiologist blinded to both PET and histopathological response data measured tumor diameters on baseline and follow-up CT images.

After scanning, the mice were anesthetized and ocular blood samples (0.2 mL) were collected before the mice were sacrificed. Tumor tissue samples were collected and the tumor inhibitory rate was calculated using the following formula:

$$\begin{array}{l} \text{Tumor}\;\text{inhibitory}\;\text{rate}\;\left(\%\right)\\ \;=(1-\text{treatment}\;\text{group}\;\text{quality}/\\ \;\text{control}\;\text{group}\;\text{quality})\times 100\% \end{array}$$

### VEGF biomarker assays

Mouse blood samples were collected using serum separator tubes and were allowed to clot for 30 minutes prior to centrifugation at 2000 g for 15 minutes. Serum samples were immediately stored at −80°C. Vascular endothelial growth factor (VEGF) levels were measured in duplicate for each serum sample using a commercially available rat quantitative sandwich enzyme immunoassay kits (ELISA) according to the manufacturer’s (Boster, Wuhan, Hubei, China) instructions. For mouse serum analysis, VEGF levels were measured using specific mouse ELISA kits (R&D Systems, Boster, Wuhan, China). All biomarker assays were performed by blinded observers.

### Statistical methods

The Statistical Package for Social Sciences Version 17.0 (SPSS inc., Chicago, IL, USA) statistical analysis software was used for all statistical analyses of the results, and data are expressed as mean±Standard deviation (SE). Two-tailed t-tests and one-way analysis of variance (ANOVA) with a Bonferroni correction for inter-group comparisons were used. A P value of <0.05 was used to denote significant difference.

## Results

### Autoradiography

**Figure 1:**
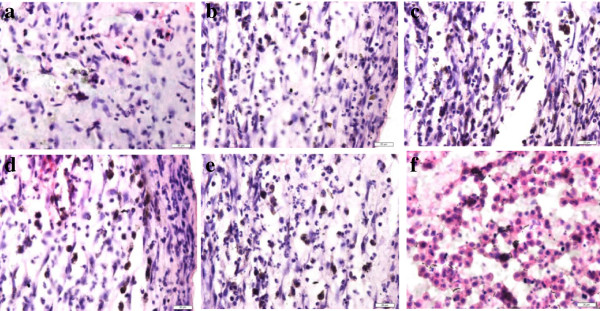
**Autoradiography (ARG) results for tumor tissue (HE×100). a**: 0.5 hour, **b**: 1 hour, **c**: 2 hour, **d**: 4 hour, **e**: 8 hour, **f**: 72 hour.

For both the test and control groups, tumors were detected at the local injection site, with the tumor formation rate is 100%. For the ARG groups, black silver particles were distributed in the tumor cell in all the slices (see Figure 1) at different time points. At 4 hours, the mean density of silver particles in the tumor tissue was low and increased gradually. At 8 hours, the mean density of the silver particles was the highest among the groups. At 72 hours, an obvious decrease in the mean silver particle density was observed. Through the ARG, we observed that the silver particles were mainly distributed in the cytoplasm and cell nuclei; however, some were also distributed in the cell membrane.

### Distribution of ^131^I-His-IN in mice

**Figure 2:**
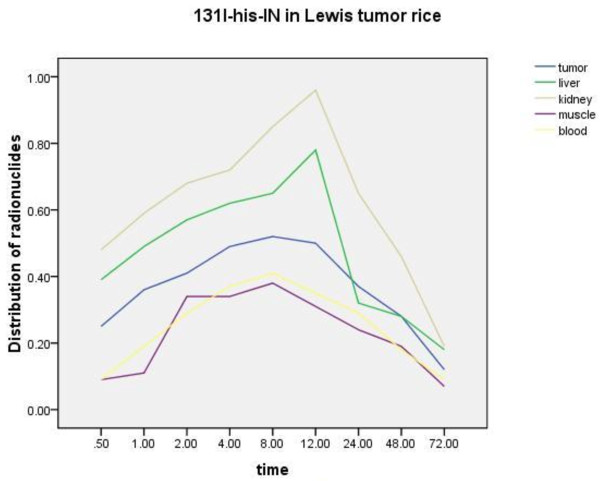
^**131**^**I-His-IN distribution in different organs.**

Then from the in vivo distribution results and tumor/non tumor (T/NT) ratio in mice bearing Lewis-lung cancer cells, it showed that ^131^I-His-IN was mostly distributed in the blood, liver, kidney, and tumor tissue (see Figure 2). Eight hours later, the distribution of radioactivity in tumor tissue reached its peak (52%). The radioactivity distribution in extra-tumor tissue then decreased gradually. The distribution of radioactivity in tumor tissue, liver, kidneys, muscle, and blood differed significantly (F=3.46, P<0.05).

### ^18^F-FDG PET-CT Imaging

**Figure 3:**
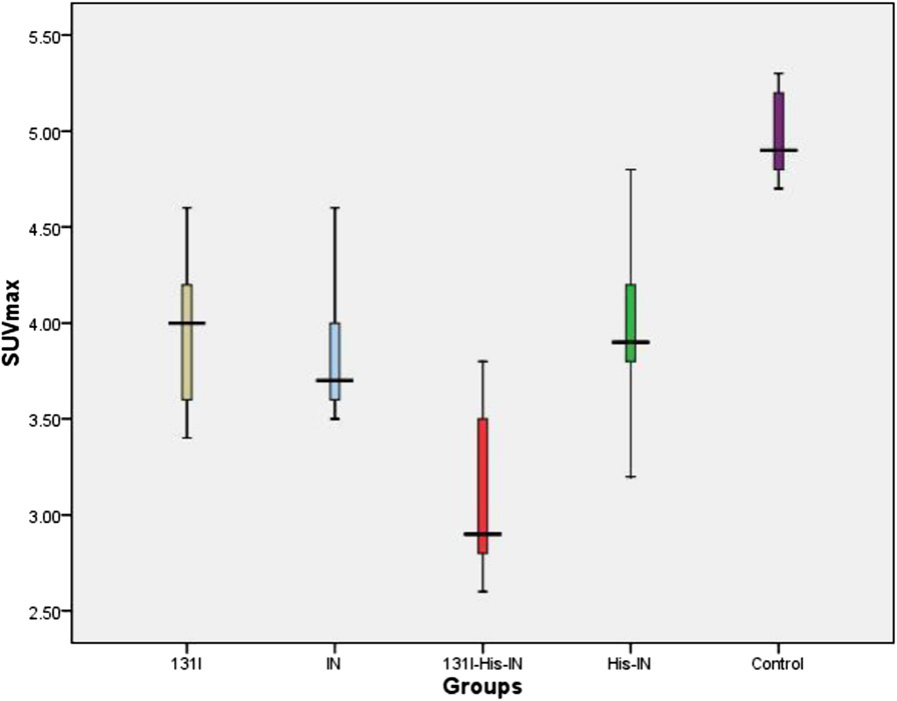
^**18**^**F-FDG positron emission tomography-computed tomography (PET-CT) maximum standard uptake value (SUVmax) in treatment groups.**

Before treatment, the ROI of tumor tissue SUVmax was not different significanty (4.3 ± 1.3). After different drugs’ treatment, the SUVmax of the tumor tissue in test groups (from K to N groups) was reduced (3.73 ± 0.57) compared with the control (4.85± 0.38). The difference was statistically significant (F = 6.54, P < 0.05), with ^131^I-His-IN group reduced significantly (see Figure 3). The rest groups (^131^I group, IN group, His-IN group) were not different greatly.

### Tumor inhibitory rate

Table 1 showed the variation in tumor volume between the treatment groups and the control group of the Lewis-bearing lung cancer mice during treatment. The tumor growth for all groups was slow during treatment after 7 days of treatment; growth inhibition was significant (p<0.05) in the treatment groups compared with the control. All treated groups showed a suppression of Lewis tumor growth, especially for the^131^I-His-IN group.

**Table 1:** Weight and tumor inhibitory rates of tumors in mice

**Groups**	**Tumor weight/g**	**Tumor volume**	**Tumor inhibitory rate(%)**
Control	4.3±1.43	5.84±1.25	
^131^I^*^	3.74±0.57	3.68±0.84	27.1^*^
IN	3.51±0.62	3.45±0.47	30.4
His-IN	3.48±0.79	3.61±0.34	34.5
^131^I-His-IN	2.79±0.41	2.08±0.63	54.8

*^131^Iodine was injected into tumor tissue location.

### VEGF level in ELISA

After ^131^I-His-IN was give the VEGF levels between the 0.5-, 48-, and 72-hour groups were significantly different (P<0.05). After different treatments, VEGF levels decreased significantly compared with the control group (F=7.74,P<0.05), especially for the ^131^I-His-IN and His-IN groups.

### Pathology

**Figure 4:**
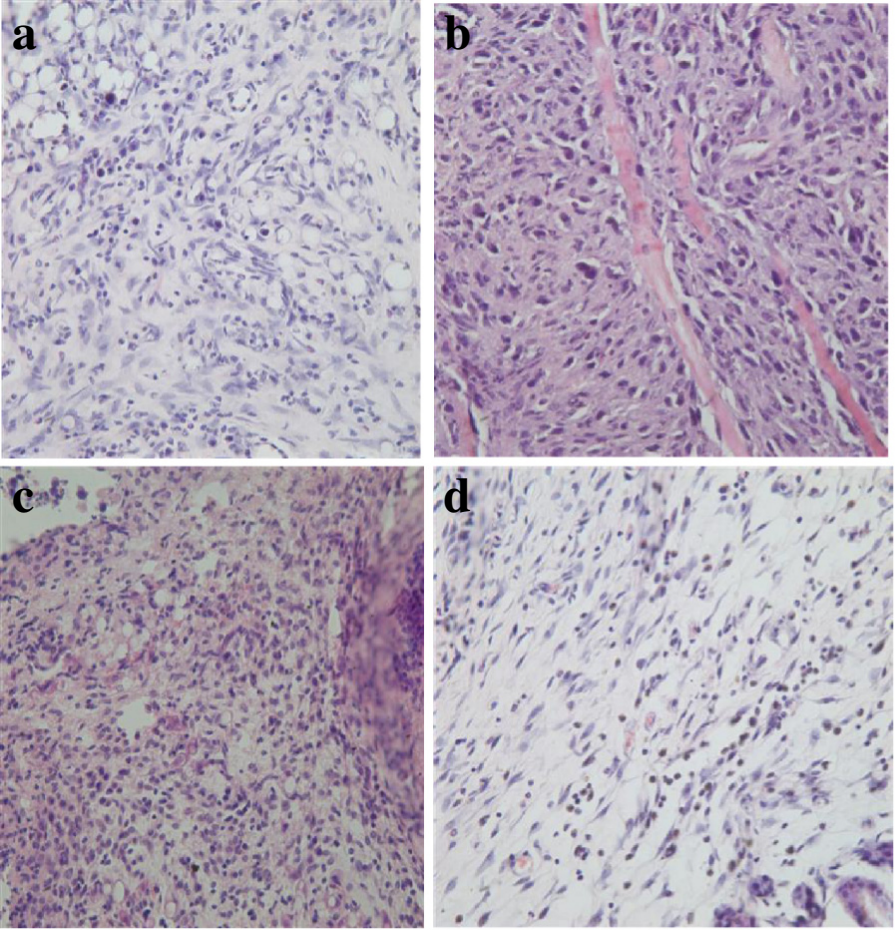
**Tumor tissue in HE**pathology**at 4 hours after treatment (HE×40). a**: Control, **b**: ^131^I, **c**: His-IN, **d**: ^131^I-His-IN.

**Figure 5:**
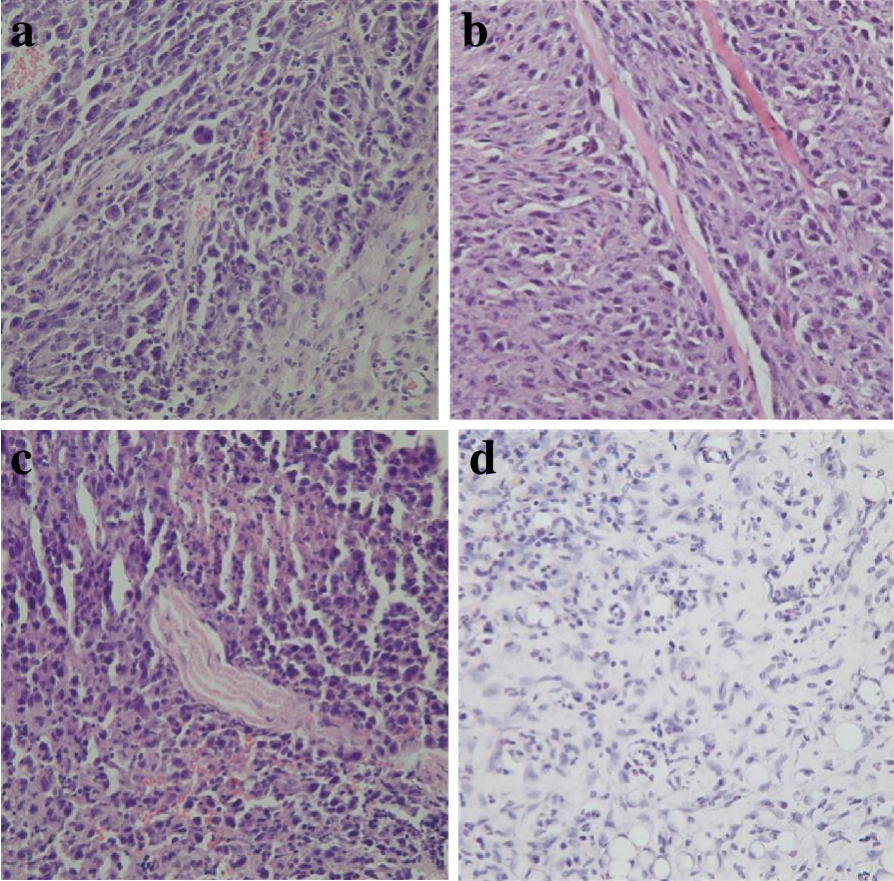
**Tumor tissue in HE**pathology**at 8 hours after treatment (HE×40). a**: Control, **b**: ^131^I, **c**: His-IN, **d**: ^131^I-His-IN.

**Figure 6:**
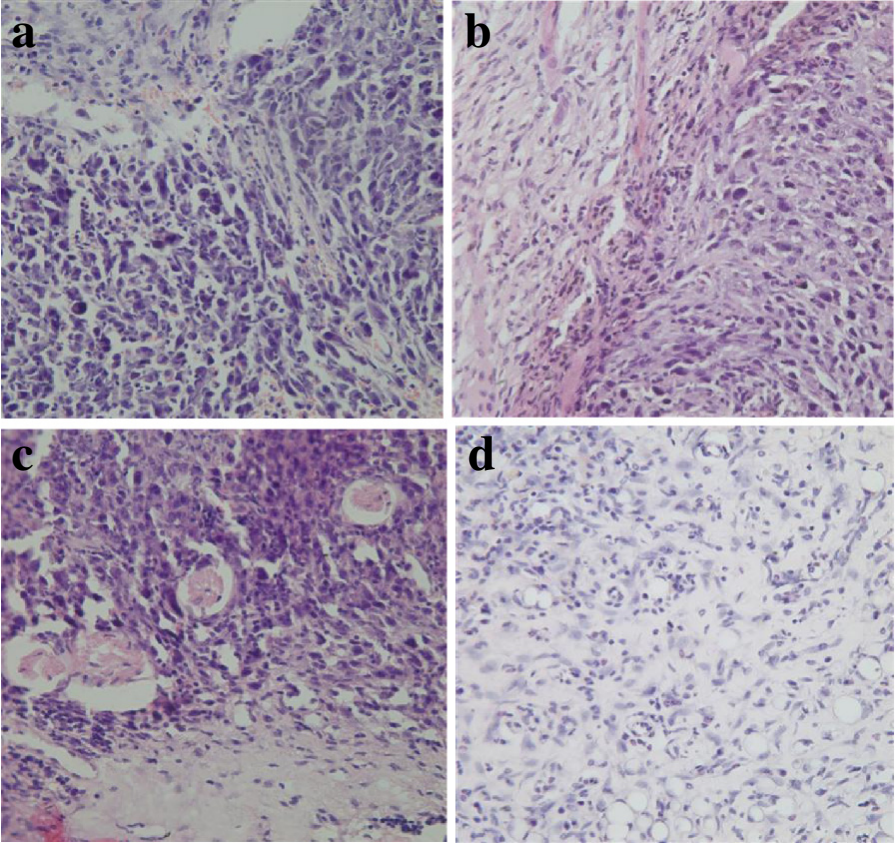
**Tumor tissue in HE**pathology**at 72 hours after treatment (HE×40). a**: Control, **b**: ^131^I, **c**: His-IN, **d**: ^131^I-His-IN.

The diameter of tumors in mice bearing Lewis lung cancer tumors was between 1.5 cm and 2.0 cm; In HE-dyed slices of the control group prior to treatment, the tumor tissue was round and spindle, with a rich cytoplasm and the nuclear in a circle with an obvious nucleoli, and with more nuclear division. After treatment, necrosis of the center of the tumor tissue gradually increased, especially for the ^131^I-His-IN group (Figure 4, 5, 6).

## Discussion

In recent years, A few research have reported that indomethacin has a sensitization and drug-resistant role [2-5]. At the same time, indomethacin may affect the tumor cell growth cycle, preventing increasement and inducing apoptosis. It could update immune status and stimulate the body to produce tumor necrosis factor (TNF), interleukin (IL)-2, and interferon(INF) factors. Thus, it could have both a direct anti-tumor effect and assist in anti-tumor functions of other agents [6-8].

With the development of nuclear imaging technology, ARG has played an important role in the understanding of the effects of radioactive drugs at the cellular and sub-cellular levels. It played an important role in researching the metabolism and toxicology evaluations. It could also assist in designing a clinical treatment plan. In addition to its role for radioactive isotopes [9], ARG also has biological characteristics, which can be used to observe the quantitative distribution and exact positioning of the cell, according to its biological metabolism. LiKun Zhou [10] et al applied tritium-labeled indomethacin and found indomethacin could focus on the cell membrane, cytoblastema, and nucleus. Our study used ^131^I-His-IN in mice, with the ARG slices, finding the drug targeted at the cell membrane, cell cytoplasm, and the nucleus. The drug mainly distributed in the cytoplasm and nucleus. This result was similar to an earlier report [10]. Research by ChunMei Chen [11] showed that indomethacin can penetrate the bi-layer of the cell membrane structure into tumor cells in the cytoplasm and even the nucleus. Based on this finding, indomethacin may also have an anti-tumor role in the membrane, cytoplasm, and the nucleus. In the cytoplasm, indomethacin may target the mitochondria directly, affecting its energy metabolism or through oxidative phosphorylation to affect the mitochondrial indirectly.

Other research [12] has shown that indomethacin may work on drug resistance by inducing apoptosis, influencing the energy metabolism of the mitochondria, especially oxidative phosphorylation. This latter effect may increase the activity of oxygen by blocking the oxidative phosphorylation, since the activity of oxygen was an important factor in suppressing tumor growth. Jiu bo Cai [13] reported indomethacin could reverse lung cancer cell drug resistance by improving ^99^ Tc^m^ intake A549 /DDP and inhibit MDRL and Pg-P expression.

The data of our research indicated that ^131^I-His-IN was mostly distributed in the blood, liver, kidney, and tumor tissue. At 8 hours, the distribution of radioactivity in tumor tissue reached its peak (52%). The distribution of radioactivity were differently among organs. At 72 hours, the radioactive count was reduced significantly, and this may be related to the short physical and biological half-life of ^131^iodine. Kanfeng Liu [14] also reported that ^131^I-His-IN was absorbed in the stomach and then absorbed and stored in tumor tissues and excreted via the kidney.

In our study, in PET-CT groups, the SUVmax was greater than 2.5 (at 4.3 ±1.3) for the tumor tissue location before treatment. After 7 days of treatment, 2 days later the mice underwent ^18^ F-FDG PET-CT scanning again, ^18^F-FDG uptake of the ROI in the tumor site decreased greatly. The SUVmax of the treated groups (3.73 + 0.58) were lower than the control group (5. 3 + 0.43).

Our data suggested the SUVmax among the treatment groups reduced differently, with ^131^ I-His-IN decreasing significantly (F = 6.54, P < 0.05). This showed that ^131^I-His-IN inhibited tumor cells may through both chemotherapy and radiation. The possible mechanisms may include: indomethacin can gather specific nuclide into tumor cells or on its surface as the carrier. At the same time ^131^I emits nuclear rays (**β**particles) aimed at tumor tissue, killing the cancer cells; Indomethacin may inhibit tumor chemical toxicity and enhance the nuclide ionizing radiation effect. In the future, further study may fucus on radiation-chemical-biological therapy complexes, which would provide triple therapy to the tumor locally. In addition, ^131^I could release γ rays, which comprise single-photon emission computed tomography (SPECT) imaging for tumor tissue [14].

The half-life of ^131^I is 8.06 days, which is helpful for transportation and clinical application. In the procession of decay, β、γ rays may be released, whose mainly energy at 610 keV and 364 keV spectively. Indomethacin could carry ^131^I and aim it in the tumor site for a long time, which preventing the damage to normal tissue or other organs greatly, If ^131^ I-Hi-IN could be made into anti-tumor targeting probe, which will become a hot point for radiation therapy research.

Serum VEGF could be used in diagnosis, evaluation before surgery, and monitoring of effective treatment and prognosis for lung cancer. Angiogenesis is the growth and metastasis of cells and the anatomical and physiological basis for lung cancer. To inhibit angiogenesis is key in controlling invasion and metastasis of cancer cells and this inhibition could improve the prognosis and survival rates for patients. Studies [15,16] have confirmed that VEGF has a high expression in non-small cell and small cell lung cancers. TianGang Xie et al [17] confirmed a high level of expression of VEGF in peripheral blood for lung cancer patients using ELISA methods. The preoperative level was statistically different compared with a normal control group. Yang Dong Xia et al [18] research showed that VEGF mainly expressed in the cytoplasm of lung cancer cells, it showed a clear heterogeneity. It had a close relationship with tumor angiogenesis. In our research, in ^131^I-His-IN group, VEGF contents decreased gradually over time. Statistical significance was seen among the different timepoints (F = 5.48, P < 0.05), especially at 0.5 and 48 hours (P < 0.05). VEGF levels were reduced in the treatment groups compared with the control group (F = 7.74, P < 0.05), with ^131^I-His-IN and His-IN groups decreased greatly. These findings indicated that ^131^I-His-IN may have an antitumor role by reducing VEGF level.

## Conclusions

We observed that ^131^I-His-IN selectively accumulated in the tumor tissue through ARG. ^131^I-His-IN was focused on the nucleus and cytoplasm. It could reduce VEGF expression and increase tumor inhibitory rates. It contributed to necrosis of the tumor tissue. ^131^I-His-IN may improve anti-tumor functions in the Lewis lung cancer. ^18^F-FDG PET-CT could be used for curative effects and for monitoring the prognosis of lung cancer.

## Abbreviations

131I-His-IN: ^131^iodine-labeled histamine-indomethacin; ARG: Autoradiography; ROI: Region of interest; HE: Hematoxylin and eosin; 18F-FDG: ^18^F- fluorodeoxyglucose; PET-CT: Positron emission tomography-computed tomography; PET: Positron emission tomography; CT: Tomography computed; IN: Indomethacin; His-IN: Histamine-indomethacin; SUVmax: Maximum standard uptake value; VEGF: Vascular endothelial growth factor; NSAID: Nonsteroidal anti-inflammatory drug; 131I: Iodine^131^; OSEM: Ordered Subset Expectation Maximization; ELISA: Enzyme immunoassay kits; SE: Standard deviation; T/NT: tumor/non tumor; TNF: Tumor necrosis factor; IL-2: Interleukin-2; INF: Interferon; SCLC: Small-cell lung cancers; SPECT: Single-photon emission computed tomography; VEGFR: Vascular endothelial growth factor receptors.

## Competing interests

The authors declare that they have no competing interests.

## Authors’ contributions

GL; CZ; GZ designed and planned the experiments. GL, RL and CC performed the experiments. GL; CZ; GZ evaluated the data and wrote the manuscript. All co-authors read and approved the manuscript.
